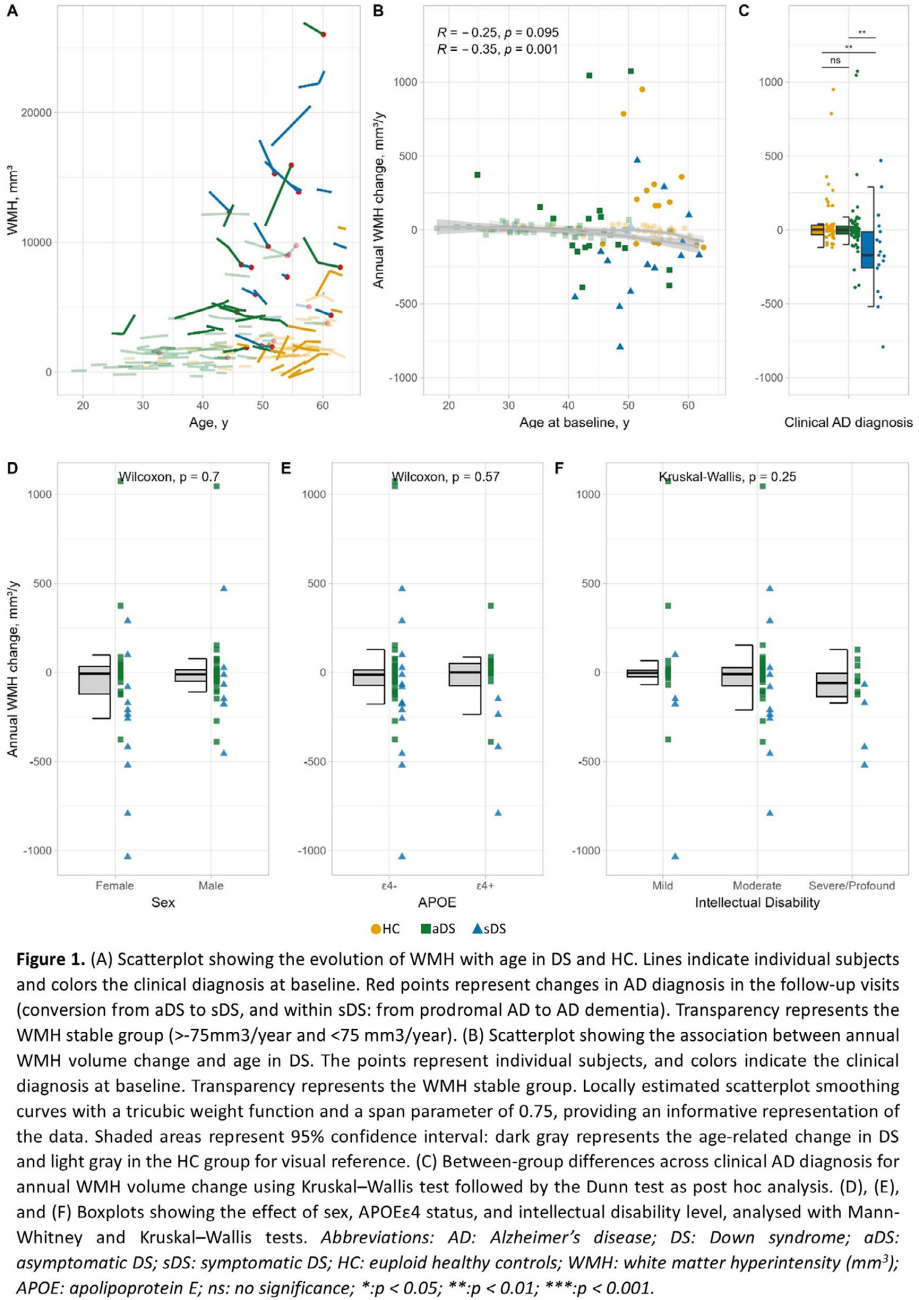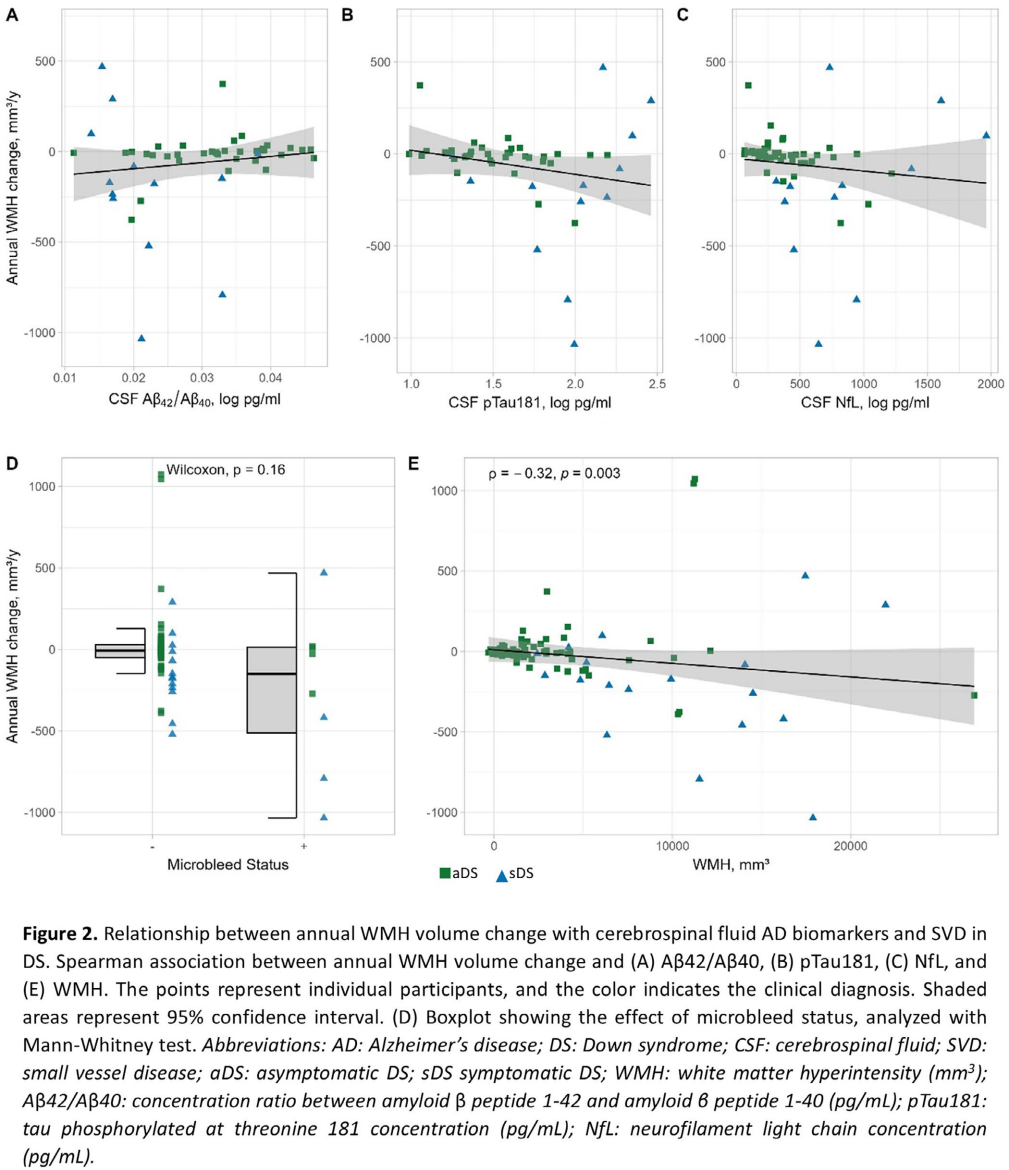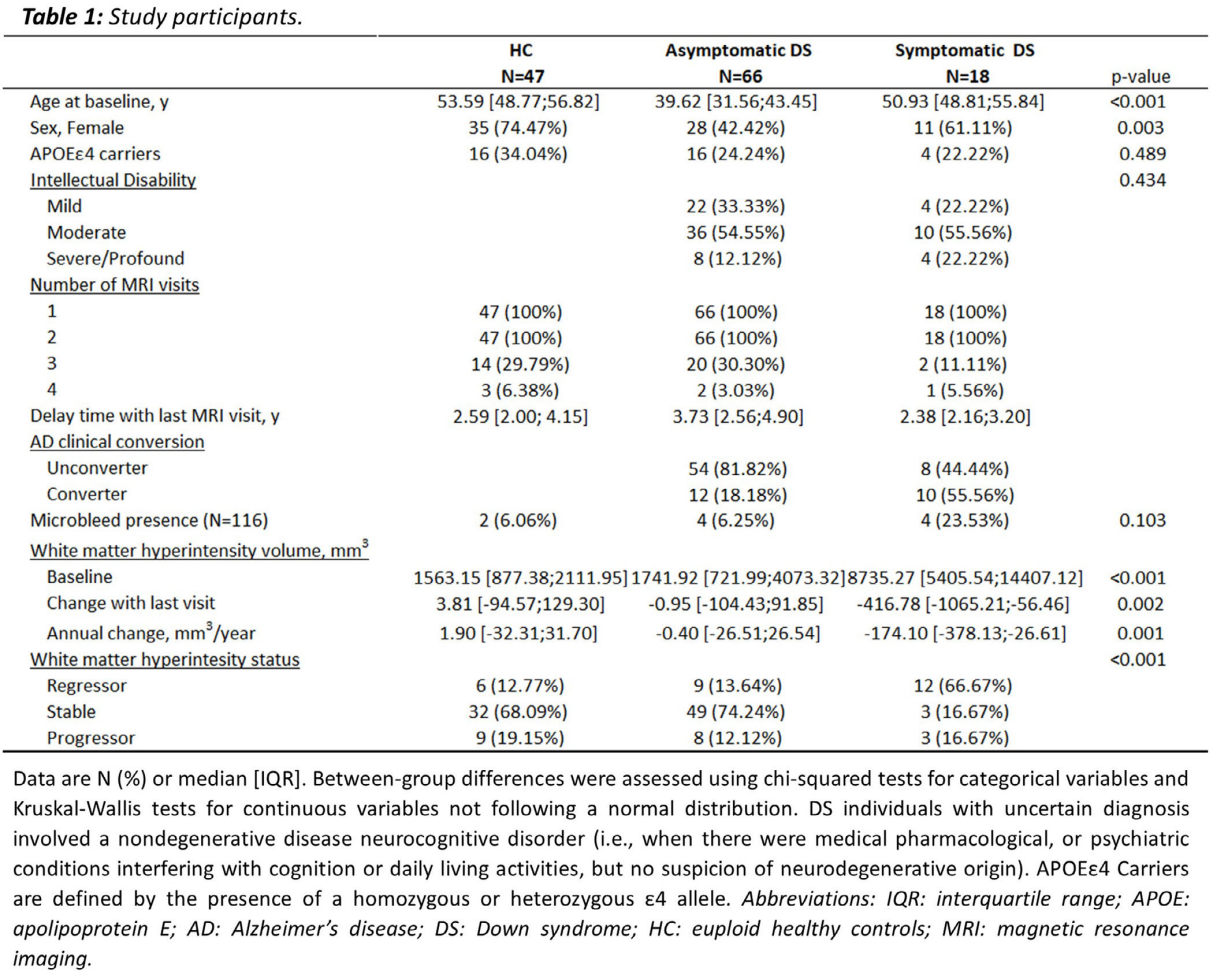# Unveiling the Temporal Dynamics of White Matter Hyperintensities Related to Alzheimer's Disease in Down Syndrome

**DOI:** 10.1002/alz70856_106414

**Published:** 2026-01-09

**Authors:** Alejandra O. Morcillo‐Nieto, Mateus Rozalem Aranha, José Enrique Arriola‐Infante, Maria Franquesa‐Mullerat, Sara E Zsadanyi, Lídia Vaqué‐Alcázar, Jose Allende Parra, Zili Zhao, Javier Arranz, Íñigo Rodríguez‐Baz, Lucía Maure‐Blesa, Laura Videla, Isabel Barroeta, Laura Del Hoyo, Bessy Benejam, Susana Fernandez, Aida Sanjuan Hernandez, Sandra Giménez, Daniel Alcolea, Olivia Belbin, Alberto Lleó, Maria Carmona‐Iragui, Juan Fortea, Alexandre Bejanin

**Affiliations:** ^1^ Center of Biomedical Investigation Network for Neurodegenerative Diseases (CIBERNED), Madrid, Spain; ^2^ Sant Pau Memory Unit, Hospital de la Santa Creu i Sant Pau, Biomedical Research Institute Sant Pau, Universitat Autònoma de Barcelona, Barcelona, Spain; ^3^ Neuroradiology Section, Department of Radiology, Hospital de la Santa Creu i Sant Pau, Biomedical Research Institute Sant Pau, Universitat Autònoma de Barcelona, Spain, Barcelona, Spain; ^4^ Department of Medicine, Faculty of Medicine and Health Sciences, Institute of Neurosciences, University of Barcelona, Barcelona, Spain. Institut d’Investigacions Biomèdiques August Pi i Sunyer (IDIBAPS), Barcelona, Spain; ^5^ Barcelona Down Medical Center, Fundació Catalana Síndrome de Down, Barcelona, Spain; ^6^ Multidisciplinary Sleep Unit, Hospital de la Santa Creu i Sant Pau, Barcelona, Spain; ^7^ Sant Pau Memory Unit, Hospital de la Santa Creu i Sant Pau, Biomedical Research Institute Sant Pau, Universitat Autònoma de Barcelona, Barcelona, Cataluña, Spain; ^8^ Sant Pau Memory Unit, Hospital de la Santa Creu i Sant Pau, Biomedical Research Institute Sant Pau, Barcelona, Spain

## Abstract

**Background:**

Down syndrome (DS) is a genetically determined form of Alzheimer's disease (AD) characterized by a low prevalence of traditional age‐related vascular risk factors. Emerging evidence indicates that white matter hyperintensities (WMH) are frequent in DS and linked to small vessel disease and neurodegeneration. However, the temporal dynamics of WMH and their relationship with AD pathology remain unexplored in DS. Using an optimal longitudinal preprocessing pipeline, we aimed to determine the evolution of WMH across the AD continuum in DS and define their associations with baseline clinical and pathological characteristics.

**Method:**

Longitudinal study including 47 euploid healthy controls (HC) from the SPIN cohort, and 86 individuals with DS from the DABNI cohort, who underwent 2 to 4 3T‐MRI visits (Table 1). The DS cohort included individuals with asymptomatic (aDS, *n* = 66) and symptomatic AD (sDS; *n* = 18). WMH were segmented on high‐resolution FLAIR images using the longitudinal pipeline from the Lesion Segmentation Toolbox in SPM12. Individuals were classified as WMH Regressor, Stable, and Progressor using a previously proposed threshold (±75 mm^3^/year, Al‐Janabi et al., 2019). Non‐parametric tests assessed the effect of sociodemographic and genetic factors, AD clinical stage, cerebrospinal fluid (CSF) AD biomarkers (Aβ42/Aβ40 ratio, pTau181, and NfL), WMH volume, and microbleed status on annual WMH volume changes.

**Result:**

WMH volume significantly decreased with age in DS (rho=‐0.37, *p* <0.001; Figure 1A‐B) but not in HC (*p* = 0.1). This decrease was more pronounced in sDS than aDS (Figure 1C). Regressor individuals were significantly more frequent in sDS (66.67%) than aDS (13.64%) or HC (12.77%). Sex, APOEε4 status, intellectual disability, and microbleed status did not influence WMH changes (Figure 1D‐E‐F). CSF‐pTau181 and NfL, but not Aβ42/Aβ40 ratio, related to annual WMH changes (Figure 2A‐B‐C). Additionaly, higher baseline WMH volume correlated with decreasing WMH changes (rho=‐0.33, *p* <0.002, Figure 2E). Sensitivity analyses considering white matter atrophy confirmed the robustness of our findings.

**Conclusion:**

WMH decreased more frequently over time in DS compared to the general population, particularly in individuals with symptomatic AD and high baseline WMH volume. This unexpected finding, which cannot be attributed to atrophy, offers novel insights into WMH aetiology in DS and has significant implications for targeted interventions using WMH as an outcome.